# Discrepancies in ejection fraction measurements between echocardiography and cardiovascular magnetic resonance lead to different clinical classifications

**DOI:** 10.1186/1532-429X-15-S1-O11

**Published:** 2013-01-30

**Authors:** Florian Andre, Sebastian Buss, Cihan Celik, Hassan Abdel-Aty, Maria Fernanda Braggion Santos, Hugo A Katus, Henning Steen

**Affiliations:** 1 Department of Cardiology, University of Heidelberg, Heidelberg, Germany; 2 School of Medicine of Ribeirao Preto University of Sao Paulo, Sao Paulo, Brazil

## Background

The left ventricular (LV) ejection fraction (EF) is a crucial parameter for the diagnosis and therapeutic management of heart failure. Due to its wide availability and its comprehensive use, echocardiography (EC) is the standard method for the assessment of the LV function in clinical routine. As fundamental clinical decisions, e.g. initiation of medical heart failure therapy or implantation of an ICD, are based on the EF, methods like the Simpsons or Teichholz formulas have been developed for its quantification in EC. Cardiovascular magnetic resonance (CMR) is the gold-standard for the evaluation of cardiac function but comparative data between CMR and EC is scarce. Therefore, we sought to compare the agreement of functional EC and CMR measurements in a daily routine clinical setting.

## Methods

We included 1017 subjects (736 male, 281 female) retrospectively in this study who presented to our cardiology department. EC was performed on four different systems (Philips IE 33, GE Vivid 7, GE Vivid I, GE Vivid S5) and images were analyzed by experienced readers. EF was measured applying the Simpson and the Teichholz formula. CMR imaging was performed on a 1.5T whole-body MRI using a standard SSFP-sequence and short-axis views covering the LV were obtained for the EF quantification. The patients were assorted into four groups according to their respective CMR-EF (I≥55%, II=45-54%, III=35-44%, IV<35%) and the agreement with the EC-guided classification was assessed. P<0.05 was regarded as significant.

## Results

The average time period between EC and CMR was 1.45 (0-9) days. Mean EF differed significantly between CMR and both EC methods in group I and IV. The EC-EF was lower in group I and higher in group IV than CMR-EF. Furthermore, differences between CMR and the Simpson formula in group II yielded significance. Comparing the Simpson to the Teichholz formula, only the difference in group I was significant. Regarding the assortment of patients according to their EF measured by EC into the EF groups validated by CMR, there was a remarkable discrepancy in all groups (see Figure 2).

**Figure 1:**
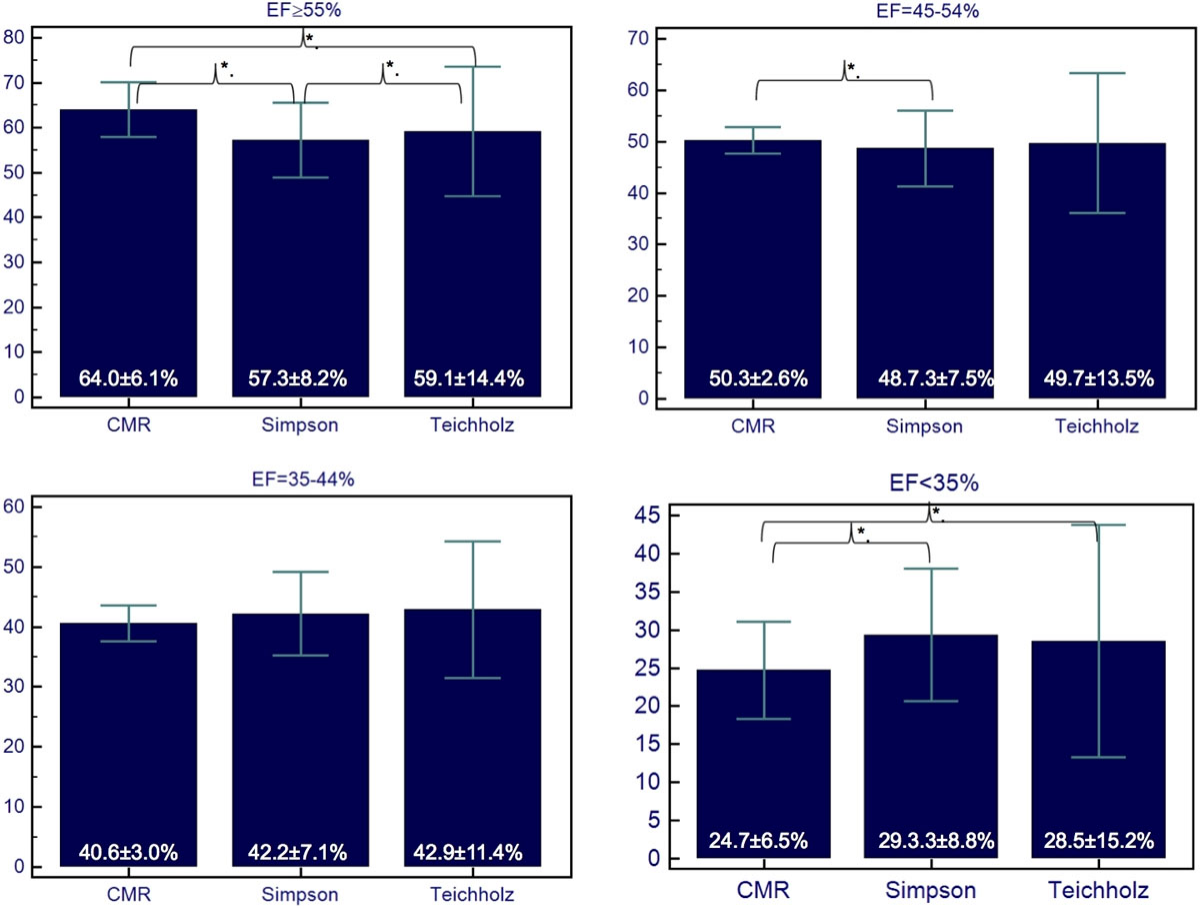
*p<0.05.

**Figure 2:**
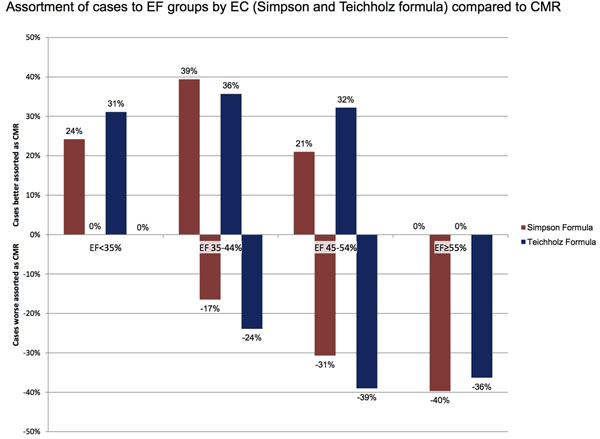


## Conclusions

The EF quantification by EC leads to a higher fraction of patients diagnosed with an impaired LV function compared to CMR who may therefore receive a medical therapy. Regarding patients with moderate or severe impaired EF, EC yields higher values assorting patients in better functional groups what may results in fewer ICD implantations in these patients. As these differences between both modalities have therapeutic consequences further studies concentrating on borderline cases are needed.

## Funding

None.

